# Pancreatic Leiomyosarcoma With Schistosomiasis Hematobia: A Case Report and Literature Review

**DOI:** 10.3389/fonc.2021.638905

**Published:** 2021-03-31

**Authors:** Qiang Li, Daniel Staiculescu, Yurong Zhou, Jiang Chen

**Affiliations:** ^1^ Department of Radiology, The Affiliated People’s Hospital of Ningbo University, Ningbo, China; ^2^ Department of Radiation Oncology, Massachusetts General Hospital, Harvard Medical School, Boston, MA, United States; ^3^ Department of Radiology, Zhongnan Hospital of Wuhan University, Wuhan, China; ^4^ Department of General Surgery, Sir Run Run Shaw Hospital, Zhejiang University, Hangzhou, China

**Keywords:** schistosomiasis hematobia, imaging, metastasis, differential diagnosis, pancreatic leiomyosarcoma

## Abstract

Pancreatic leiomyosarcoma (PL) is a very rare, malignant neoplasm with a very poor prognosis. Here, we examine a novel case of PL with schistosomiasis hematobia. The patient had been initially misdiagnosed by the first magnetic resonance imaging (MRI). The second imaging examination demonstrated an enlarged heterogeneous tumor mass in the body-tail of pancreas. Following image analysis, the patient underwent a pancreatectomy, splenectomy and lymph node dissections. Sixteen months after the tumor resection, follow-up computed tomography (CT) and MRI revealed tumor metastasis in the liver and lung. PL has non-specific clinical manifestations and imaging characteristics, making early diagnosis very challenging. When it is difficult to distinguish between benign and malignant pancreatic lesions, short-term imaging follow-up is preferred. In this case report, we discuss the relationship between PL and schistosomiasis hematobia.

## Introduction

Pancreatic leiomyosarcoma (PL) is a very rare, malignant neoplasm occurring in the pancreas, accounting for 0.1% of tumors primarily arising from pancreas (1–3). It is thought to arise from nearby the pancreatic duct or pancreatic blood vessels (4). The prognosis of pancreatic leiomyosarcoma is very poor, with a 5-year mortality rate of 77.8% (4–6). Here, we report a case of PL with schistosomiasis hematobia.

## Case Presentation

**Figure 1:**
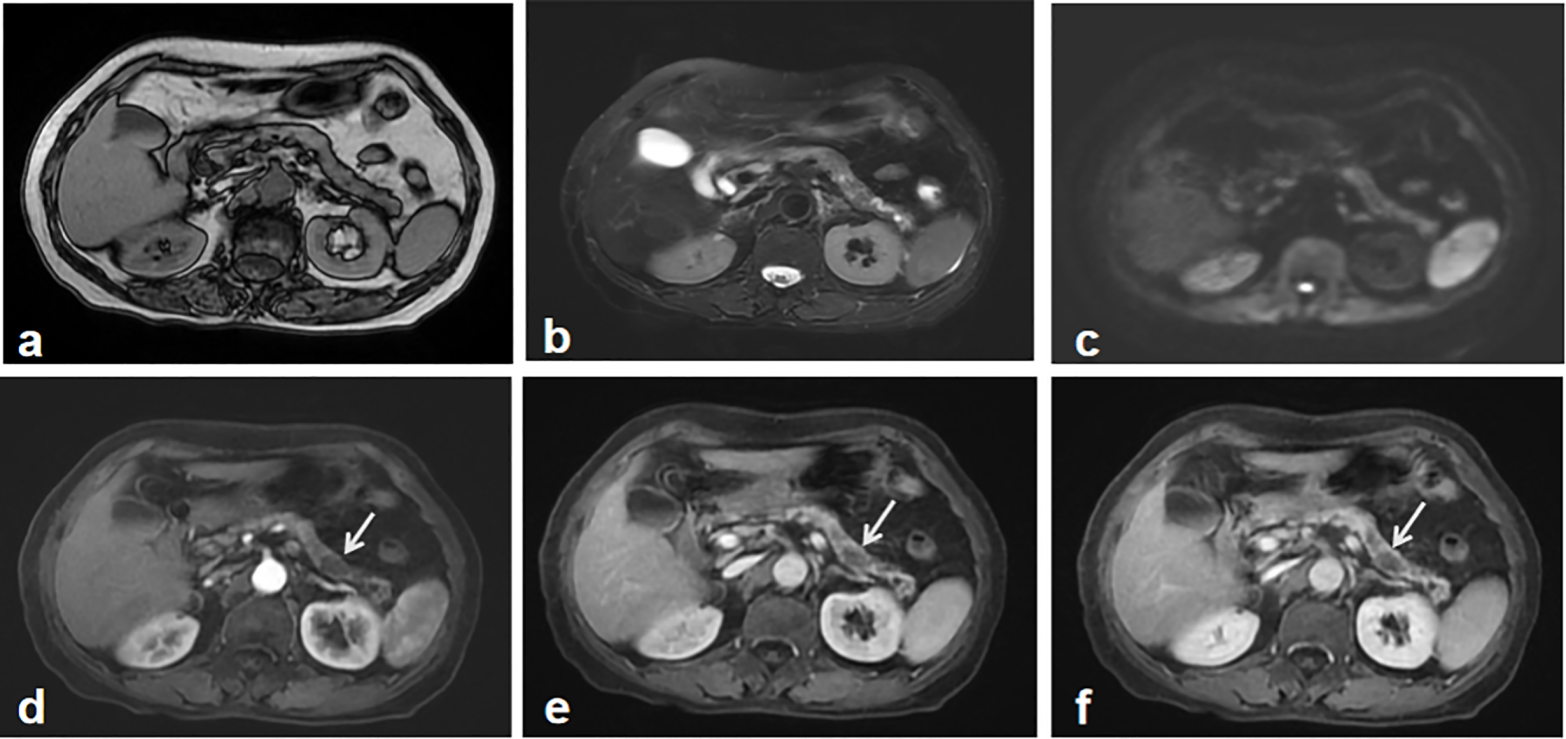
The first abdominal MRI. Both MR T1 **(A)** and T2 weighted image **(B)** showed the atrophy of the pancreas and inhomogeneous signal change in the body and tail of the pancreas. Diffusion weighted image didn’t show the apparent tumor mass-related signal change **(C)**. Gadolinium-enhanced T1 weighted images including early arterial phase **(D)**, late arterial phase **(E)** and portal phase images **(F)**, depicted multiple irregular hypointense signals without demonstrating a typical tumor imaging characteristics, which was regarded as an indeterminate lesion in the pancreas. A recommendation of imaging follow-up was made for the patient.

**Figure 2:**
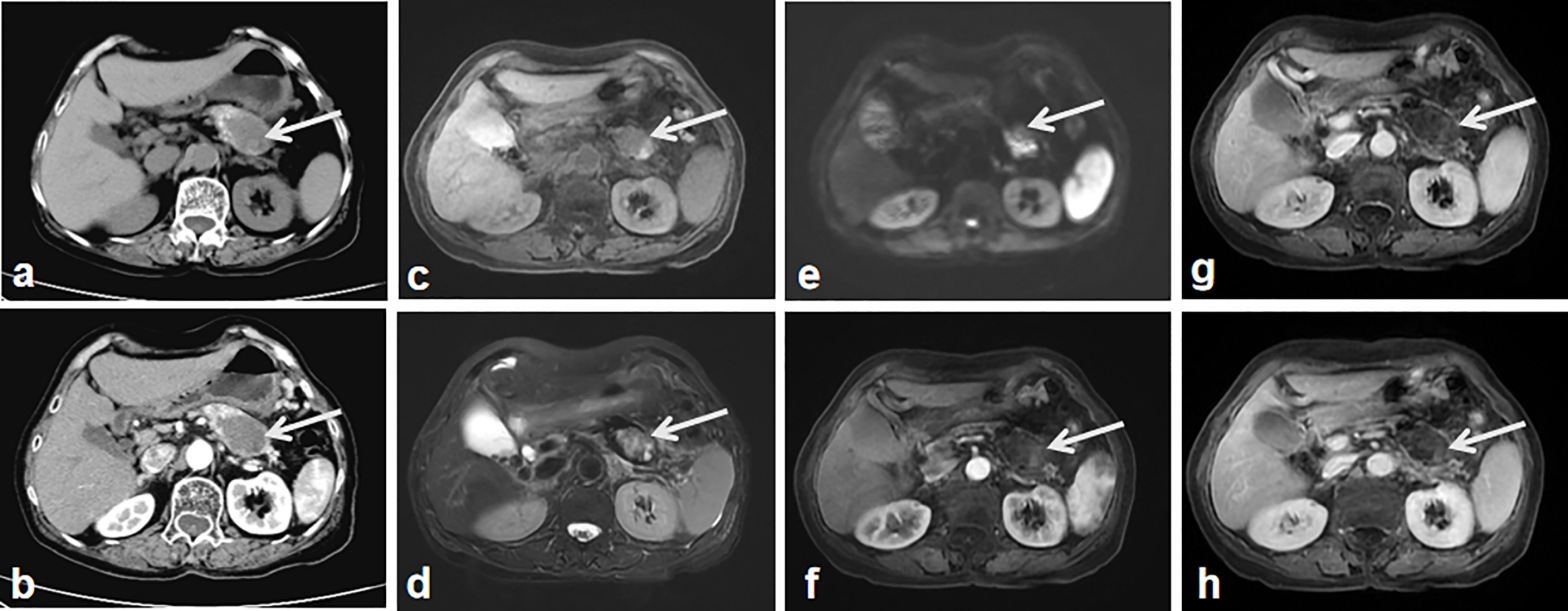
The second imaging. CT without contrast administration show the significantly enlarged tumor mass with heterogeneous components of central cystic low attenuation, peripheral solid isointensity and small patchy calcification (**A**, arrow). Contrast-enhanced CT shows the tumor mass with intermediate enhancement of the peripheral component and the non-enhanced cyst-like central area (**B**, arrow). MR T2 **(C)** and T2 weighted images **(D)** show heterogeneous hyper-signal intensity and hypo-signal intensity in the central area of the tumor (arrow). DWI shows the apparent diffusion restriction-induced heterogeneous hypersignal intensity (**E**, arrow). Contrast-enhanced MR weighted images of arterial **(F)**, portal **(G)** and equilibrium phase **(H)** show the tumor mass with intermediate enhancement of the peripheral component, the non-enhanced cyst-like central area (arrows).

**Figure 3:**
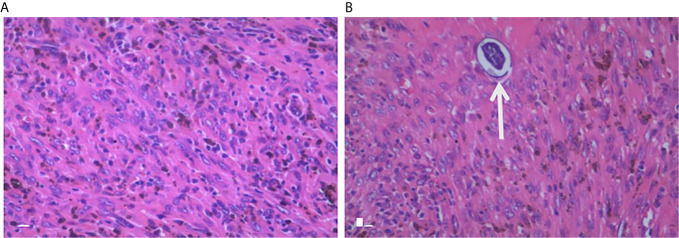
Pathological examination. Histology with H&E staining of the excised pancreatic tumor mass shows large pleomorphic, spindle-shaped cells, fibrosis, which indicate a stromal malignancy **(A)**. Another slide of the tumor shows schistosome in the tumor tissue (**B**, arrow).

**Figure 4:**
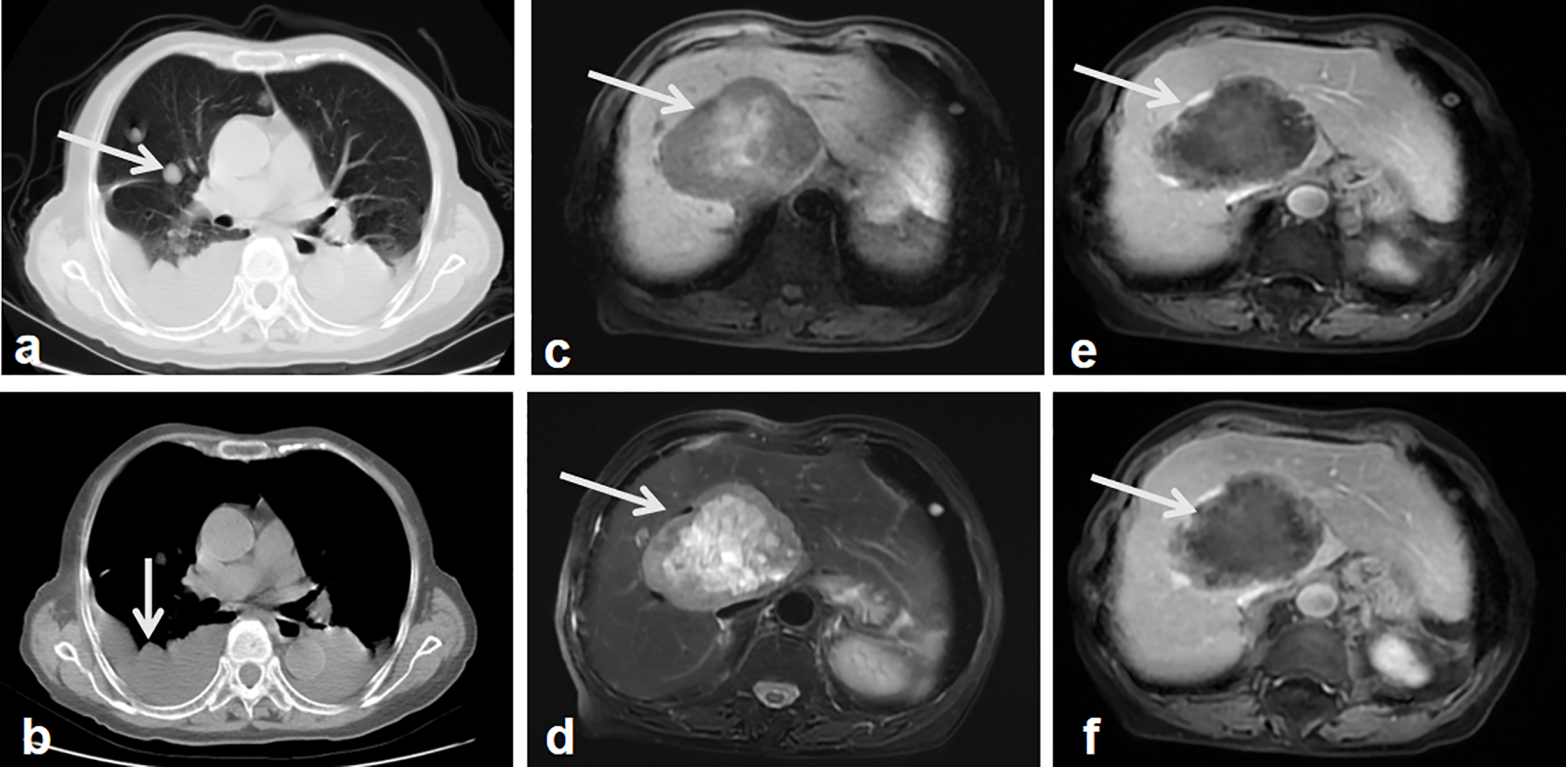
Follow-up images after surgery. Lung CT shows multiple nodules in the lung and thoracic effusion, which indicate the lung and possible pleural metastasis (**A, B**, arrow). MR T1 weighted images show a well-demarcated, heterogeneous hypointense signal tumor in the right lobe of the liver with patchy hypersignal intensities in the central of the tumor (**C**, arrow). MR weighted images show the tumor mass with heterogeneous hypersignal intensities (**D**, arrow). Contrast-enhanced MR weighted images of arterial **(E)**, portal phase **(F)** show the tumor mass with intermediate enhancement of the peripheral component, the non-enhanced hypointense signal central area (arrows).

A geriatric female, of over 70 years, presented epigastric pain in April 2013. The patient underwent MRI, which revealed abnormal signal changes in the body of pancreas, the atrophy of the pancreatic tail, dilation of distal pancreatic duct, and less enhancement than the pancreatic parenchyma (**Figure 1**). It is difficult to tell if the pancreatic lesions are benign, premalignant, or malignant through this imaging alone. Thus, an imaging follow-up was recommended. In November 2014, the ultrasound imaging follow-up showed a significantly enlarged mass, which was confirmed by computed tomography (CT) and MRI examination. CT also revealed a tumor mass in the body-tail of pancreas, demonstrating that the mass contains mixed cyst-like and solid components as well as peripheral patchy calcification on plain CT. Mild enhancement of the solid components and non-enhancement of the central necrosis were also present (**Figures 2A, B**). MRI showed inhomogeneous hyperintensity on T2 weighted images, restricted diffusion on diffusion-weighted images, and mild inhomogeneous enhancement of the solid components on the gadolinium-enhanced T1 weighted images (**Figures 2C–H**). Laboratory testing showed increased serum ferritin (280.90 ng/ml), while other tumor markers (such as alpha-fetoprotein (α-AFP), carcino-embryonic antigen (CEA), carbohydrate antigen 19-9 (CA19-9), and carbohydrate antigen 125 (CA-125)) were normal. Since no metastases were found, a pancreatic tumor resection was performed. A 5 × 5 cm tumor mass was found in the body-tail of the pancreas with enlarged lymph nodes, surrounding the hepatic artery, in the laparotomy. Adjacent to the pancreatic tumor mass, another 2 × 2 cm tumor was found adhering to the splenetic vein. After the tumor resectability was thoroughly assessed, the patient underwent a pancreatectomy with a splenectomy and regional lymph node dissection. Histological analysis showed that the mass located in the pancreatic body and tail had focal necrosis. The diagnosis of PL was confirmed by H&E morphology and immunohistochemical staining (**Figure 3A**). Interestingly, few dead *Schistosoma* eggs were found in the tumor tissues (**Figure 3B**), and a large number of eosinophils surrounded the *Schistosoma* eggs. Twenty months after surgery the patient developed liver and lung metastasis (**Figure 4**), and she died three months later.

## Discussion

PL is a rare malignant neoplasm, first reported by Ross in 1951 (2). Fewer than 100 cases of PL have been documented in the literature (7). The mean age of patients is 53.9 ± 14.7 years old (range = 14–87 years) with an equal incidence rate between men and women (3, 7). The most common symptoms of PL include abdominal pain, weight loss, epigastric tenderness, and abdominal mass formation (8, 9). The size of the tumors is quite variable (from 3 to 25 cm) with median size of 10.5 cm (4, 10). Patients that present large tumors also display hemorrhagic, necrotic changes, which are usually associated with a highly aggressive behavior and can be misdiagnosed as a pseudocyst or cystadenocarcinom in early stage by imaging. The only potentially curative approach is surgery before widespread metastasis.

What were the imaging features of PL with *Schistosoma japonicum*, and why it was misdiagnosed by first MRI? PL is characterized by a large heterogeneous mass with peripheral enhancement *via* CT scanning while MRI scanning shows hyperintensity of a mass on T2 weighted images with peripheral enhancement (7, 11, 12). In the above case, the main imaging characteristic was a heterogeneous mass with a cystic component due to necrosis at pancreatic body and tail. These image signs were similar when compared with previous PL reports, but many of these same features can also be found in pancreatic pseudocysts (13, 14), mucinous cystic neoplasms (13, 15), intraductal papillary mucinous tumor(IPMT) (16), solid and papillary epithelial neoplasm (SPEN) (17, 18), and pancreatic neuroendocrine tumors (19). Since there are no specific imaging features unique to just PL and the substantial overlap of the population and symptoms compared with other pancreas malignant tumors, an accurate differential diagnosis is difficult to obtain. A cystic lesion of the pancreas, on first MRI, was misdiagnosed in the above case without further examination, such as a PET/CT or biopsy.

Was PL induced by *S. japonicum* or was primary malignant tumor accompanied with parasitic infection? The host–parasite interactions during schistosomiasis induce a Th1/Th2 response by a subset of immune response genes, such as IL-4, IL-6, and IL-10) (20). *S. japonicum* is a chronic infection where regulatory pathways accommodate host permissiveness to schistosome establishment of productive parasitism (20, 21). Associated carcinogenesis might be induced, by frequent reinfections, due to increased proliferation, angiogenesis, mutagenesis, and oncogene activation (22, 23). While Schistosomiasis-associated malignant tumors might also be induced by activated macrophages and leukocytes, many mechanisms remain unclear (1, 22, 24). There were a few scattered neutrophils surrounding Schistosoma eggs without microphages in our presented case, so schistosomiasis may increase PL progression.

In summary, PL has non-specific clinical manifestations and imaging characteristics, but shares similar imaging appearance with many other pancreatic neoplasms. When it is difficult to distinguish between benign and malignant pancreatic lesions, other examinations (such as PET/CT, biopsy) and short-term imaging follow-up are preferred. The relationship between schistosomiasis and PL might be not relevant, but more clinical evidence is needed to confirm this assertion.

## Data Availability Statement

The raw data supporting the conclusions of this article will be made available by the authors, without undue reservation.

## Ethics Statement

Written informed consent was obtained from the individual(s) for the publication of any potentially identifiable images or data included in this article.

## Author Contributions

QL and YZ took the lead in drafting the manuscript and provided magnetic resonance images and H&E staining. DS, YZ, and JC provided supervision and participated in the literature review and in drafting the manuscript. All authors contributed to the article and approved the submitted version.

## Funding

This work was supported by Public Welfare Technology Research Program of Zhejiang Natural Science Foundation (grant number: LGF20H180005 to QL), China Postdoctoral Science Foundation Funded Project (grant number: 2020T130584 to JC), and China Scholarship Council (grant number: 201806325017, to JC). This work was also supported by Wuhan University Training Program for Young Talents Abroad (grant number 2018-105 to YZ).

## Conflict of Interest

The authors declare that the research was conducted in the absence of any commercial or financial relationships that could be construed as a potential conflict of interest.

## Abbreviations

PL, Pancreatic leiomyosarcoma; MRI, magnetic resonance imaging; CT, computed tomography; α-AFP, alpha-fetoprotein; CEA, carcino-embryonic antigen; CA19-9, carbohydrate antigen 19-9; CA-125, carbohydrate antigen 125; IPMT, Intraductal papillary mucinous tumor; SPEN, solid and papillary epithelial neoplasm.

## References

[1] Bacelar A Castro LG de Queiroz AC Cafe E. Association between prostate cancer and schistosomiasis in young patients: a case report and literature review. Braz J Infect Dis (2007) 11:520–2. 10.1590/S1413-86702007000500014 17962880

[2] Ross CF. Leiomyosarcoma of the pancreas. Br J Surg (1951) 39:53–6. 10.1002/bjs.18003915311 14858827

[3] Reyes MC Huang X Bain A Ylagan L. Primary pancreatic leiomyosarcoma with metastasis to the liver diagnosed by endoscopic ultrasound-guided fine needle aspiration and fine needle biopsy: A Case Report and Review of Literature. Diagn Cytopathol (2016) 44:1070–3. 10.1002/dc.23540 27455910

[4] Hur YH Kim HH Park EK Seoung JS Kim JW Jeong YY. Primary leiomyosarcoma of the pancreas. J Korean Surg Soc (2011) 81 Suppl 1:S69–73. 10.4174/jkss.2011.81.Suppl1.S69 PMC3267071 22319744

[5] Muhammad SU Azam F Zuzana S. Primary pancreatic leiomyosarcoma: a case report. cases J (2008) 1:280. 10.1186/1757-1626-1-280 18957130 PMC2584078

[6] Aleshawi AJ Allouh MZ Heis FH Tashtush N Heis HA. Primary Leiomyosarcoma of the Pancreas: a Comprehensive Analytical Review. J Gastrointest Cancer (2020) 51:433–8. 10.1007/s12029-019-00282-1 31392629

[7] Fadaee N Sefa T Das A Rajkomar K. Pancreatic leiomyosarcoma: a diagnostic challenge and literature review. BMJ Case Rep (2019) 12:e231529. 10.1136/bcr-2019-231529 PMC6887380 31780603

[8] Hebert-Magee S Varadarajulu S Frost AR Ramesh J. Primary pancreatic leiomyosarcoma: a rare diagnosis obtained by EUS-FNA cytology. Gastrointest Endosc (2014) 80:361–2. 10.1016/j.gie.2014.02.030 PMC4154627 25034846

[9] Machado MC Cunha JE Penteado S Bacchella T Jukemura J Costa AC. Preoperative diagnosis of pancreatic leiomyosarcoma. Int J Pancreatol (2000) 28:97–100. 10.1385/IJGC:28:2:097 11128979

[10] Milanetto AC Lico V Blandamura S Pasquali C. Primary leiomyosarcoma of the pancreas: report of a case treated by local excision and review of the literature. Surg Case Rep (2015) 1:98. 10.1186/s40792-015-0097-2 26943422 PMC4595416

[11] Riddle ND Quigley BC Browarsky I Bui MM. Leiomyosarcoma arising in the pancreatic duct: a case report and review of the current literature. Case Rep Med (2010) 2010:252364. 10.1155/2010/252364 20589089 PMC2892659

[12] Makimoto S Hatano K Kataoka N Yamaguchi T Tomita M Nishino E. A case report of primary pancreatic leiomyosarcoma requiring six additional resections for recurrences. Int J Surg Case Rep (2017) 41:272–6. 10.1016/j.ijscr.2017.10.039 PMC5681329 29121584

[13] Cohen-Scali F Vilgrain V Brancatelli G Hammel P Vullierme MP Sauvanet A. Discrimination of unilocular macrocystic serous cystadenoma from pancreatic pseudocyst and mucinous cystadenoma with CT: initial observations. Radiology (2003) 228:727–33. 10.1148/radiol.2283020973 12954892

[14] Sahani DV Kadavigere R Saokar A Fernandez-del Castillo C Brugge WR Hahn PF. Cystic pancreatic lesions: a simple imaging-based classification system for guiding management. Radiographics (2005) 25:1471–84. 10.1148/rg.256045161 16284129

[15] Freeny PC Saunders MD. Moving beyond morphology: new insights into the characterization and management of cystic pancreatic lesions. Radiology (2014) 272:345–63. 10.1148/radiol.14131126 25058133

[16] Lim JH Lee G Oh YL. Radiologic spectrum of intraductal papillary mucinous tumor of the pancreas. Radiographics (2001) 21:323–37; discussion 337-40. 10.1148/radiographics.21.2.g01mr01323 11259696

[17] Buetow PC Buck JL Pantongrag-Brown L Beck KG Ros PR Adair CF. Solid and papillary epithelial neoplasm of the pancreas: imaging-pathologic correlation on 56 cases. Radiology (1996) 199:707–11. 10.1148/radiology.199.3.8637992 8637992

[18] Mortele BP Mortele KJ Tuncali K Banks RA Glickman J Silverman SG. Solid and papillary epithelial neoplasm of the pancreas: MR imaging findings. JBR-BTR (2002) 85:297–9. 12553659

[19] Sahani DV Bonaffini PA Fernandez-Del Castillo C Blake MA. Gastroenteropancreatic neuroendocrine tumors: role of imaging in diagnosis and management. Radiology (2013) 266:38–61. 10.1148/radiol.12112512 23264526

[20] Feng M Cheng X. Parasite-Associated Cancers (Blood Flukes/Liver Flukes). Adv Exp Med Biol (2017) 1018:193–205. 10.1007/978-981-10-5765-6_12 29052139

[21] Nair SS Bommana A Bethony JM Lyon AJ Ohshiro K Pakala SB. The metastasis-associated protein-1 gene encodes a host permissive factor for schistosomiasis, a leading global cause of inflammation and cancer. Hepatology (2011) 54:285–95. 10.1002/hep.24354 PMC3125413 21488078

[22] Botelho MC Vale N Gouveia MJ Rinaldi G Santos J Santos LL. Tumour-like phenotypes in urothelial cells after exposure to antigens from eggs of Schistosoma haematobium: an oestrogen-DNA adducts mediated pathway? Int J Parasitol (2013) 43:17–26. 10.1016/j.ijpara.2012.10.023 23260770

[23] Hamid HKS. Schistosoma japonicum-Associated Colorectal Cancer: A Review. Am J Trop Med Hyg (2019) 100:501–5. 10.4269/ajtmh.18-0807 PMC6402928 30560774

[24] Dizdaroglu M Olinski R Doroshow JH Akman SA. Modification of DNA bases in chromatin of intact target human cells by activated human polymorphonuclear leukocytes. Cancer Res (1993) 53:1269–72. 8383005

